# Antibody Conjugates for Sarcoma Therapy: How Far along Are We?

**DOI:** 10.3390/biomedicines9080978

**Published:** 2021-08-08

**Authors:** Letizia Polito, Giulia Calafato, Massimo Bortolotti, Cecilia Chiarelli Olivari, Stefania Maiello, Andrea Bolognesi

**Affiliations:** Department of Experimental Diagnostic and Specialty Medicine-DIMES, General Pathology Section, Alma Mater Studiorum—University of Bologna, 40126 Bologna, Italy; giulia.calafato2@unibo.it (G.C.); massimo.bortolotti2@unibo.it (M.B.); cecilia.chiarelli@studio.unibo.it (C.C.O.); stefania.maiello2@unibo.it (S.M.); andrea.bolognesi@unibo.it (A.B.)

**Keywords:** sarcoma, cancer therapy, immunoconjugates, immunotherapy, antibody, drug delivery, ribosome-inactivating proteins, bacterial toxins, radionuclides

## Abstract

Sarcomas are one of the most difficult type of cancer to manage and treat because of their extremely heterogeneous molecular and morphological features. Despite the progress made over the years in the establishment of standard protocols for high and low grading/staging sarcoma patients, mostly with chemotherapy and/or radiotherapy, 50% of treated patients experience relapse episodes. Because of this, in the last 20 years, new therapeutic approaches for sarcoma treatment have been evaluated in preclinical and clinical studies. Among them, antibody-based therapies have been the most studied. Immunoconjugates consist of a carrier portion, frequently represented by an antibody, linked to a toxic moiety, i.e., a drug, toxin, or radionuclide. While the efficacy of immunoconjugates is well demonstrated in the therapy of hematological tumors and more recently also of epithelial ones, their potential as therapeutic agents against sarcomas is still not completely explored. In this paper, we summarize the results obtained with immunoconjugates targeting sarcoma surface antigens, considering both preclinical and clinical studies. To date, the encouraging results obtained in preclinical studies allowed nine immunoconjugates to enter clinical trials, demonstrating the validity of immunotherapy as a promising pharmacological tool also for sarcoma therapy.

## 1. Introduction

Sarcomas are a heterogeneous-low-incidence group of malignancies that arise from mesenchymal tissue. They comprehend more than 50 histotypes with different molecular biology, epigenetic landscape, and variable response to treatments. Although sarcomas can develop anywhere in the body, they are found mostly in the arms, legs, chest, and abdomen. According to their tissue-origin, sarcomas are classified in two major groups: soft tissue sarcoma (STS) and bone sarcoma (BS).

In 2021, American Cancer Society’s estimates show that about 3610 new cases of BS and 13,460 of STS will be diagnosed, with 2060 and 5350 deaths expected, respectively [1]. Epidemiology data indicate that sarcomas have not the same incidence in all age groups, but it is possible to identify two peaks in people <20 years and 65–74 years. For younger people, the percentage of incidence, compared to total sarcoma cases, is 25.1% (BS) and 7.5% (STS). For elder people, this percentage decreases to 13.2% for BS while increases up to 20.3% for STS [2].

The main therapy for sarcoma treatment is surgery accompanied by neoadjuvant or adjuvant chemotherapy/radiotherapy. Standard sarcoma drugs mostly include doxorubicin and ifosfamide, but according to the histological type, cancer staging and grading several other drugs can be used [3].

Over the years, many progresses have been made on STS patients with localized disease at diagnosis, achieving a 5-year relative survival rate of 81.3%. Unfortunately, this rate dramatically drops to 16% in patients with metastasized STS at diagnosis. The 5-year relative survival of patients diagnosed with bone and joint cancer is 66.8% [2]. Approximately 50% of patients with high-grade STS experienced relapse, progression and metastasis after the first-line standard treatment [4]. These data and the heterogeneous nature of sarcomas support the idea that using a personalized therapy instead of a standardized protocol could be a valid strategy to improve the patient’s outcome.

Immunotherapy is one of the most promising individualized therapeutic approaches for the treatment of cancer that uses immune system components to fight the disease. Over the years, many clinical trials have reported the effects of antibody-based therapies on a variety of tumors, including sarcoma, in terms of improved overall survival compared to conventional chemotherapy drugs.

## 2. Immunoconjugates for Targeted Cancer Therapy

Many studies have been conducted to assess the efficacy of monoclonal antibodies (mAbs) in targeted cancer therapy. The specificity of immunotherapy depends on the surface antigen expression of target cells and its cytotoxicity is independent from the parameters that determine the toxicity of chemotherapy and radiotherapy. The selected antigens should have precise characteristics: easy accessibility, high expression on targeted malignant cells, and low or no expression on non-target healthy cells. The main cytotoxic pathways that can be activated after mAbs-antigen binding are: complement-dependent cytotoxicity (CDC) and antibody-dependent cellular cytotoxicity (ADCC), mediated by the Fcγ receptors on effector cells, such as granulocytes, macrophages, and natural killers. Moreover, some antibodies can directly kill the target cell by triggering the apoptotic pathway. However, the antibody cytotoxicity is often limited because of phenomena of CDC and ADCC resistance or selection of apoptosis resistant tumor clones. Two main strategies can be adopted to overcome these obstacles, thus enhancing mAbs efficacy. First, mAbs can be used in combination with standard chemotherapy or administered to patients with highly responding cancer subtypes [5,6]. Second, antibodies can be linked to pharmacologically active molecules, combining the antibody specificity to the therapeutic effects of such molecules. This concept paved the way for the development of immunoconjugates (ICs), which contain anticancer drugs, toxins (from plants or bacteria), or radionuclides. ICs have been evaluated in numerous preclinical studies and in various clinical trials, either administered individually or in combination with conventional chemotherapy [7,8,9]. ICs are composed of three elements: a carrier molecule (i.e., an antibody or its fragment), a toxic payload, and a linker. After binding to the targeted antigen, the IC is internalized and the toxic payload can exert its pharmacological effect [10]. Choosing an antigen and an antibody should satisfy certain rules. The targeted antigen must be extracellularly exposed and expressed higher on cancer cells rather than healthy ones. The antibody should have high affinity and avidity toward antigen and efficient internalization after binding [11,12].

Various anticancer molecules have been considered for antibody–drug conjugate (ADC) production. The most used agents are distinguished into: (i) DNA-targeting drugs, which lead to DNA alkylation or double-strand break (i.e., duocarmycins, calicheamicins, pyrrolobenzodiazepines, anthracycline, and camptothecin derivatives) and (ii) tubulin-targeting drugs, which block tubulin depolymerization, thus determining cell-cycle arrest into G2/M phase (i.e., monomethyl auristatin E and F, MMAE and MMAF, respectively) [13,14]. Four requirements are essential in addressing the drug choice: potency, stability, water solubility, and easy conjugation. It is crucial to find a balance between drug toxicity, generally with effective concentrations in the nM range, and in vivo systemic tolerability. Moreover, the stability in blood circulation and water solubility of the molecule are necessary to guarantee a proper distribution in body fluids. Lastly, the molecular and chemical structure of the drug should allow the conjugation with the linker, thereby facilitating drug-linker binding to the antibody [15].

In addition to common anticancer drugs, plant or bacteria toxins can be used in IC construction. Ribosome-inactivating proteins (RIPs) are plant toxins able to deadenylate rRNA, thus irreversibly blocking protein synthesis and inducing cell death. Beside ribosomes, RIPs can act on other substrates such as DNA, mRNA, tRNA, and poly(A), whose damage causes the activation of multiple cell death pathways (i.e., apoptosis, necroptosis) and oxidative stress [16,17]. Bacterial toxins are other powerful tools that can be used as payload in IC construction. The most used ones are *Pseudomonas aeruginosa* exotoxin A (PE) and diphtheria toxin, and their truncated forms, which arrest protein synthesis by inactivating the elongation factor 2 through ADP-ribosylation. RIPs or bacterial toxins can be conjugated to an antibody (or its fragment), constituting the so-called immunotoxins (ITs). Over the years, many ITs have been constructed and tested in preclinical and clinical studies in different cancer models, showing promising results both in hematological and solid tumors [18,19]. The use of toxins rather than anticancer drugs in the construction of an IC has some advantages. Being enzymes, toxins act in a catalytic and not in a stoichiometric way as drugs do. Moreover, toxins do not induce drug resistance, a phenomenon that is often observed in patients treated with chemotherapeutics [20]. Lastly, toxins can act on both dividing and non-dividing cells, while most chemotherapeutic drugs only act on proliferating cells [21].

Radionuclides represent another type of payload type used in targeted therapy. In this case, the antibody is (radio)labeled with a radioisotope that emits ionizing particles to obtain a radioimmunoconjugate (RIC). Each particle (α and β^-^ particles and Auger electrons) is characterized by a specific linear energy transfer, physical half-life, and penetration depth in tumor tissue, thus offering different possibilities of use according to the physical characteristic of the tumor (large tumors, micro-metastasis, single cancer cells) [22,23]. Radioimmunotherapy (RIT) advantages in cancer therapy are represented by the stability and low dimension of (radio)labeled conjugates. RICs can easily reach cancer sites and kill target cells without the typical chemotherapy side effects [24,25]. RICs can act not only on target cells but also on the surrounding ones. This characteristic represents an advantage because also tumor stromal cells and cancer cells with low antigen expression, or expressing mutated antigens, will be eliminated. At the same time, this RIC property is also potentially dangerous because of its aspecific toxicity to normal tissues. Other difficulties are related to RIC manipulation and stability as well as radionuclide half-life. For this reason, it is essential in clinical practice to manage properly radiation intensity, time of exposure, and administration protocol, in order to maximize efficacy and reduce possible damages on radio-sensitive organs such as bone marrow.

**Figure 1:**
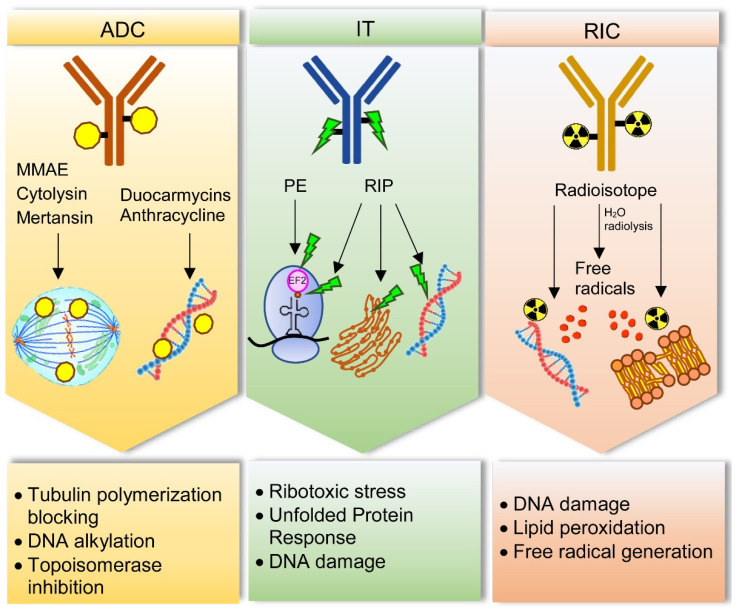
Main cell damage mechanisms induced by immunoconjugates for sarcoma treatment. Antibody–drug conjugates (ADC) can contain drugs acting with different mechanisms. Monomethyl auristatin E (MMAE), cytolysin and mertansin block tubulin polymerization, thus hampering cell cycle. Duocarmycins and anthracycline target DNA, inducing alkylation and topoisomerase inhibition, respectively. Immunotoxins (IT) can be constructed with Pseudomonas exotoxin (PE) or ribosome-inactivating proteins (RIPs). PE inhibits elongation factor-2 (EF2) through its ADP-ribosylation, thus inducing ribotoxic stress and protein synthesis blocking. RIPs can inhibit protein synthesis through rRNA-N-glycosylase activity removing a specific adenine in a stem–loop region of the main ribosomal RNA. In addition, RIPs can act on different substrates such as endoplasmic reticulum, through unfolded protein response, and DNA, by directly damage. Radioimmunoconjugates (RIC) contain radioisotopes, which can damage DNA and cause lipid peroxidation in cell membranes. This effect can occur directly or indirectly through free radicals produced by water radiolysis.

The main mechanisms by which drugs, toxins, and radionuclides can damage cancer cells are schematized in Figure 1.

To date, many clinical trials have been conducted to investigate the efficacy and safety of ICs in patients with hematological and solid cancers, administered alone or in combination with other therapeutic agents. In the last 20 years, Food and Drug Administration approved 10 ADCs, 1 IT and 2 RICs for targeted cancer therapy [26,27,28,29].

## 3. Immunoconjugates for Sarcoma Therapy

Although ICs have showed relevant effects mainly on hematological malignancies, numerous studies have paved the way to their application for solid tumors, including sarcomas. Unlike hematological tumors, sarcomas as well as all solid cancers have some molecular and morphological characteristics that make them more difficult to treat with IC-based therapy [30]. In particular, the difficulty of penetration inside tumor mass is related to the abundant extracellular matrix, disorganized vasculature and absence of functional lymphatic vessels that causes increased interstitial fluid pressure [31]. However, protocol optimization, progressive reduction of IC size and implementation of penetration efficacy are expected to significantly improve targeted therapy of solid tumors [32,33].

### 3.1. Antibody–Drug Conjugates for Sarcoma

Many studies have reported the efficacy of ADCs towards specific antigens expressed in different types of sarcoma.

The endosialin/CD248/TEM1 receptor is a transmembrane glycoprotein expressed on pericytes and fibroblasts during embryogenesis. In adults, its presence dramatically drops in normal tissues while it is expressed in mesenchymal tumors, such as sarcoma, neuroblastoma, as well as in perivascular and tumor-associated stroma [34]. Moreover, it is associated with tumor angiogenesis and inflammation [35,36]. In sarcomas, this antigen is highly expressed on the surface of malignant, perivascular, and stromal cells, even on high grade and advanced sarcoma [37,38]. Two anti-endosialin ADCs were tested in preclinical models of sarcoma. The antitumor efficacy of the anti-endosialin-MC-VC-PABC-MMAE was tested on two endosialin-positive human cell lines and one sarcoma xenograft model. Inhibiting concentration 50 (IC_50_) values were 0.5 µg/mL for the Ewing sarcoma (ES) cell line A-673 and 1.5 µg/mL for the osteosarcoma (OS) cells SJSA-1, without any correlation between the extent of cell growth inhibition and endosialin expression levels. The antitumor activity of this ADC was also tested in nude mice bearing A-673 cells xenografts. The dose of 15 mg/kg of anti-endosialin-MC-VC-PABC-MMAE determined a marked and durable inhibition of tumor growth leading to mice survival of 80% after day 150, thus demonstrating the antitumor efficacy of this ADC [39]. The anti-endosialin ENDOS/ADC was tested in sarcoma preclinical models. This IC is composed of a humanized anti-endosialin mAb hMP-E-8.3 linked to a duocarmycin derivative alkylating agent. In this case, SJSA-1 cells resulted more sensitive to ENDOS/ADC than A-673 cells, displaying IC_50_ values of 0.8 nM and 8.6 nM, respectively. Moreover, in a SJSA-1 derived xenograft model, mice treated with ENDOS/ADC showed a strong reduction of tumor volume [40].

Glycoprotein non-metastatic b (GPNMB) is a transmembrane protein involved in bone differentiation and remodeling [41,42]. Different cancer cell types are characterized by high levels of this glycoprotein, among them OS cells [43,44]. In addition, GPNMB is involved in cancer migration, invasion, progression, and metastasis, as well as poor patient prognosis [45,46]. The fully human IgG2 mAb CR011, which recognizes GPNMB extracellular domain, was linked to MMAE in the Glembatumumab vedotin ADC. This IC was tested in preclinical models of OS: 10 short-term cell cultures from patient-derived OS, 5 standard OS cell lines and 4 xenograft cell lines. Glembatumumab vedotin had a significant cytotoxic activity, displaying IC_50_ values lower than 55 μg/mL in most of the treated cells. Moreover, ADC effect correlates with GPNMB expression levels [47]. Glembatumumab vedotin entered phase 2 clinical trial involving 22 patients (ranging 12–50 years) with recurrent or refractory OS. This ADC was administered intravenously at 1.9 mg/kg/dose over 90 min on day 1; the treatment was repeated every 21 days for up to 18 courses. The results showed a limited efficacy. In fact, only one patient had a partial response and two maintained a stable tumor disease. No correlation was observed between GPNMB expression and clinical response (NCT02487979).

Leucine-rich repeat containing 15 (LRRC15), a member of the Leucine-Rich Repeat superfamily, is another target evaluated for ADC-based sarcoma therapy. LRRC15 is overexpressed in cancer-associated fibroblasts and cancer cells from many epithelial and mesenchymal solid tumors. In particular, it was reported that OS tissue samples had high LRRC15 expression both on cancer and stroma cells [48]. ABBV-085 ADC is composed of the anti-LRRC15 humanized IgG1 kappa antibody Ab1 conjugated to the antimitotic drug MMAE. The antitumor efficacy of ABBV-085 was evaluated in a cancer+/stromal+ patient-derived xenograft (PDX) of OS. Results showed that ABBV-085 was extremely effective in terms of tumor growth inhibition, in comparison to other standard OS therapies (doxorubicin, ifosfamide, gemcitabine, cisplatin) [49,50]. A multicenter phase 1 dose-escalation clinical trial of ABBV-085 is currently under investigation in patients with advanced solid tumors, including undifferentiated pleomorphic sarcoma (NCT02565758).

CD56, also called Neural Cell Adhesion Molecule (NCAM), is a homophilic binding glycoprotein present on the surface of neurons and glia where it has a prominent role in neuronal adhesion and migration ability, neurite outgrowth, synapse formation and synaptic plasticity [51,52]. CD56 can also be found in hematopoietic cells, above all in natural killer cells, where it acts as an adhesion molecule [53]. CD56 is over-expressed in different cancer types, like neuroblastoma, rhabdomyosarcoma (RMS) and most of the STS, Wilms tumor, acute myeloid leukemia, glioma, and astrocytoma, as well as in several carcinomas [54,55,56]. Lorvotuzumab mertansine (IMGN901) is an ADC composed of an anti-CD56 humanized N901 mAb conjugated to the maytansinoid DM1, via a stable disulfide linker. IMGN901 was tested in vitro on two RMS and two ES cell lines showing a great sensitivity, which is not always correlated to CD56 expression intensity. In vivo studies were conducted in tumor xenograft models; stable complete responses were observed in two out of seven RMS xenografts; even in this case there was not a strong correlation between CD56 expression levels and treatment response. The response variability might be due to factors other than CD56 expression, such as mitotic rate, chemoresistance to tubulin targeting agents and/or intracellular processing of IMGN901 [57]. This conjugate in a phase 1 trial on myeloma patients had demonstrated ample evidence of safety and signals of clinical activity [58]. These results paved the way for a phase 2 clinical trial where IMGN901 was evaluated in patients with relapsed or refractory Wilms tumor, RMS, neuroblastoma, pleuropulmonary blastoma, malignant peripheral nerve sheath tumor, or synovial sarcoma (SS). Patients received lorvotuzumab mertansine intravenously at 110 mg/m^2^ over 1–1.5 h on days 1 and 8; the treatment was repeated every 21 days for up to 17 courses in the absence of disease progression or unacceptable toxicity. Despite the high level of CD56 found in all treated pediatric tumors, only few patients had a relevant clinical response. This might be due to many factors: limited penetration of the conjugate/payload into solid tumor cancer cells, presence of unrecognized CD56 isoforms that could interfere with the binding/internalization process and tumor resistance to DM1 (NCT02452554).

Endoglin (ENG or CD105) is a homodimeric glycoprotein, expressed in endothelial cells, bone marrow cells, and macrophages, and involved in embryogenesis, angiogenesis, and vascular establishment as well as homeostasis [59,60]. In tumors its presence is associated with neo-angiogenesis, which represents a key feature of malignant cancer. ENG can be both a transmembrane protein acting as a co-receptor for transforming growth factor-β, and a soluble extracellular matrix protein after cleavage by metalloproteinase 14 occurs. In sarcomas ENG is associated with poor outcome in ES patients, being a key point in tumor cell plasticity, tumor progression and invasiveness [61]. OMTX703 is an ADC composed of the anti-ENG mAb OMTX003, which recognize ENG extracellular domain, linked to cytolysin. The antitumor efficacy of this ADC was evaluated in cell lines, cell line-derived xenografts, and PDX of ES. After OMTX703 treatment, a potent anti-proliferative effect was reported in the ES8 cell line with an IC_50_ value of 260.6 nM. In the same cell line, it was observed a correlation between ENG expression level, which was extremely high, and ADC internalization ability and cytotoxic effect. OMTX703 efficacy was assessed in a ES8 xenograft model (immunocompromised NOD-SCID-IL-2Rg^null/null^ mice), where tumor growth was strikingly reduced with a 60 mg/kg dose of ADC. Interestingly, immunohistochemistry studies confirmed that ENG expression levels in xenograft tumors were quite similar to those found in parental cell lines in vitro. Lastly, OMTX703 antineoplastic effect was evaluated in PDX models, which display the highest ENG expression and better represent the typical heterogeneity of these tumors. It was observed that OMTX703 (30 and 60 mg/kg) was able to produce a dose-dependent antitumor response. A complete response rate of 60% was achieved at the end of the treatment with the highest dose (60 mg/kg) [62].

The urokinase plasminogen activator receptor–associated protein uPARAP/Endo180 plays a crucial role in the process of collagen turnover. The receptor acts through the endocytosis of extracellular matrix collagen, which is further addressed to lysosomal degradation [63,64]. In normal tissues, this receptor is expressed in a limited range of cell types involved in tissue development, such as fibroblasts and osteogenesis-associated mesenchymal cells [65,66]. In cancer cells its expression was found to be high, especially in OS and STS. In a preclinical study the antitumor efficacy of 2h9-vc-MMAE, an ADC composed of an anti-uPARAP mAb linked to MMAE was evaluated in fibrosarcoma (FS) and RMS cell lines (HT1080 and RD, respectively), expressing high levels of uPARAP. The ADC cytotoxic mechanism is related to the binding and internalization properties as well as to ADC lysosomal cleavage. The ADC was able to significantly reduce cell viability on sarcoma cell lines even if to a lesser extent than on hematological cell lines. In addition, ADC efficacy was evaluated in mice xenografted with human leukemic cells. Complete rescue of all treated animals was observed with no sign of adverse effects [67].

Receptor tyrosine kinase-like orphan receptors (ROR) are a family of transmembrane tyrosine kinases. ROR1 is a Wnt5a receptor expressed during embryonic development and in several hematologic and solid malignancies [68]. NBE-002 is an ADC consisting of a humanized anti-ROR1 mAb conjugated to a derivative of the potent anthracycline PNU-159682 [69]. NBE-002 is currently under evaluation in a phase 1/2 clinical trial in patients (age ≥ 18 years) with advanced solid tumors, including sarcoma; NBE-002 has been given intravenously on day 1 of repeated 21-day courses (NCT04441099).

ROR2 is one of the non-canonical Wnt receptors, which plays significant roles during early embryonic development in several tissue types [70]. The protein may be involved in the early formation of chondrocytes and in osteoblastogenesis [71]. ROR2 is overexpressed during embryonic development and in several important cancer types, including sarcoma, where its levels are strongly correlated with worst prognosis of patient [70]. CAB-ROR2-ADC or BA3021 is a ROR2-targeting ADC composed of a conditionally active biologic (CAB) anti-ROR2 antibody conjugated to an undisclosed payload [72]. This ADC is currently being evaluated in a phase 1/2 clinical trial in patients (age ≥ 18 years) with locally advanced unresectable or metastatic solid tumors, including STS (NCT03504488—recruiting status).

AXL is a member of receptor tyrosine kinases TAM family. AXL is widely expressed in healthy cells and tissues, where it is involved in cell survival, phagocytic clearance of dying cells, natural killer cell differentiation, and cell aggregation [73]. AXL is also highly expressed on a variety of cancer types, OS included, where it plays a central role in tumor proliferation, survival, stem cell phenotype, metastasis, and resistance to cancer therapy [74,75]. CAB-AXL-ADC or BA3011 is composed of a CAB anti-AXL antibody conjugated to an undisclosed payload [76]. This ADC is currently under investigation in a phase 1/2 clinical trial in patients (age ≥ 18 years in phase 1; age ≥ 12 years in phase 2) with advanced solid tumors including different types of sarcoma. In phase 1 trial all patients will receive BA3011, while in phase 2 trial all patients will receive either BA3011 alone or in combination with nivolumab (NCT03425279—recruiting status). Enapotamab Vedotin or HuMax-AXL-ADC is another anti-AXL ADC, firstly tested in vitro and in vivo in preclinical models of non-small cell lung cancer [77]. It consists of a human AXL-specific IgG1 conjugated to the cytotoxic agent MMAE. Enapotamab Vedotin is now being evaluated in a phase 1/2 clinical trial in patients (age ≥ 18 years) with selected, relapsed and advanced or metastatic solid tumors, sarcoma included, which no longer respond to standard therapy (NCT02988817).

CD70 is a transmembrane antigen belonging to the tumor necrosis factor (TNF) ligand super family. Its interaction with CD27 receptor enhances cellular proliferation and induces anti-apoptotic proteins playing a major role in T-cell costimulation. CD70 is often upregulated in T- and B- cell lymphomas and in various solid tumors even if its exact role during the disease onset and progression remains unknown [78]. CD70 was identified as a specific and highly expressed surface protein in uterine leiomyosarcoma cell lines and in clinical samples of this rare and aggressive gynecologic malignancy. The antihuman-CD70 mAb vorsetuzumab was conjugated to MMAF towards uterine leiomyosarcoma cell line and its antitumor effects were evaluated in vitro and in vivo. This anti-CD70 ADC showed a significant cytotoxicity on SK-LMS-1 cells, displaying IC_50_ equal to 0.120 nM and it strongly inhibited tumor growth in SK-LMS-1 xenograft mouse models and in uterine leiomyosarcoma PDX mouse models with a relative tumor reduction of 54.5% and 84.7%, respectively [79].

ADCs tested in preclinical studies and in clinical trials are reported in Table 1.

### 3.2. Immunotoxins for Sarcoma

Various ITs have been tested for sarcoma therapy, evaluating their binding, internalization ability, and anti-tumor effect.

Chondroitin sulfate proteoglycan 4 (CSPG4) is a tumor-associated surface antigen, firstly found on human melanoma cells [80]. It is used as a marker of proliferation and metastasis in poor prognosis tumor types such as breast cancer and STS, whilst its expression is very low in healthy tissues [81]. The CSPG4-specific PE-based IT, αMCSP-ETA’, was tested for RMS adjuvant therapy. Multiple parameters were evaluated in vitro on three embryonal RMS cell lines (RD, FL-OH1 and TE-671) and one alveolar RMS cell line (Rh30). IT binding was specific on CSPG4^+^ cells and IT internalization was rapid; αMCSP-ETA’ inhibited RMS cell proliferation with IC_50_ values ranging from 0.02 to 50 nM and induced apoptosis. The binding was also evaluated ex vivo on three patient-derived paraffin-embedded RMS tumor sections, exhibiting good specificity. Although preliminary, these results highlighted the therapeutic potential of this IT (alone or combined with standard drugs) for RMS treatments [82].

The well-known epidermal growth factor receptor (EGFR) is a member of the ErbB tyrosine kinase receptor family. It is involved in routine cellular processes such as proliferation, differentiation, and cellular development [83]. EGFR is highly expressed in several solid cancers [84]. EGFR is overexpressed in up to 76% of embryonal RMS cases, so it is considered a suitable target for RMS immunotherapy [85]. The EGFR-specific recombinant IT 425(scFv)-ETA′ was tested in vitro on three different embryonal RMS cell lines RD, FL-OH1 and TE-671. Experiments demonstrated binding specificity and valuable internalization. Moreover, 425(scFv)-ETA′ was able to reduce cell viability (IC_50_ values in picomolar range) and to strongly activate apoptotic pathway. The EGFR^+^ cell binding activity of the IT 425(scFv)-ETA′ was also demonstrated ex vivo on two patient-derived formalin-fixed paraffin-embedded RMS specimens [86]. EGFR was also used as target of an indirect IT, consisting of a primary EGFR specific mAb followed by a secondary F(ab’)_2_ anti-mouse Ig linked to saporin-S6. The indirect IT caused a significant inhibition of cell growth and protein synthesis (IC_50_ 0.95 nM) and a strong increase in apoptosis in RD/18 RMS cell line. The toxic activity of the anti-EGFR IT was also observed on RMS cell lines expressing low levels of EGFR [87].

The glycoprotein gp72 is a tumor-associated cell surface antigen present in melanoma, bladder and breast carcinoma and osteogenic sarcoma [88]. 791T/36-RTA derives from the conjugation of the murine anti-gp72 mAb 791T/36 with ricin A chain (RTA) and it specifically inhibited tumor cell growth in vitro in the OS cell line 791T. The cytotoxicity of this IT depended primarily on its very rapid cell surface binding, endocytosis and intracellular processing leading to the release of the toxic payload in the cytoplasm to inhibit protein synthesis [89].

B7H3 is a cell surface glycoprotein expressed on cancer cells and not found on normal tissues [90]. It is involved in natural killer and T cell inhibition, as well as in tumor cell migration and invasion [91]. The recombinant IT 8H9(scFv)-PE38 was constructed with the truncated form of PE (PE38) conjugated to the single-chain fragment variable (scFv) of the anti-B7H3 mAb 8H9. The IT had a cytotoxic effect in vitro on three B7H3^+^ human OS cell lines (U2OS, CRL1427, and OHS-M1), with IC_50_ values of 0.03, 0.05, and 0.02 µg/mL, respectively. 8H9(scFv)-PE38 was also tested in vivo in xenograft SCID mice bearing OHS-M1 cells. The results indicated that tumor regression was achievable using 0.15 mg/kg IT without significant systemic toxicity for animals [92].

Another sarcoma-associated antigen is an 80 kDa surface glycoprotein recognized by TP-3, a mAb that particularly reacts with OS. This antigen was found to be highly expressed on OS and in some hemangiopericytoma, chondrosarcoma, malignant fibrous histiocytoma, and synovial sarcoma. Healthy tissues exhibited a very low expression of this antigen [93]. TP-3 mAb was conjugated to pokeweed antiviral protein (PAP) and the cytotoxic effect of this IT was tested in vitro on the human OS cell line OHS. TP-3-PAP was able to kill TP-3^+^ cells in a specific and efficient manner, with IC_50_ values in the picomolar range. Furthermore, the antitumor activity of this IT was assayed in vivo in a TP-3^+^ mouse model bearing human sarcoma lung metastases with good results in terms of number and size reduction of the metastases in a dose dependent manner [94]. Two recombinant TP-3 based ITs were produced combining the toxin PE38 with the monovalent and bivalent disulfide-stabilized Fv of the antibody, TP-3(dsFv)-PE38 and TP-3(dsFv)_2_-PE38, respectively. These ITs were tested in vitro on three human OS cell lines (OHS-M1, OHS, and SaOS). Results indicated a specific effect for TP-3^+^ cells, with a great binding affinity. Bivalent IT was more cytotoxic than monovalent IT, with IC_50_ of 4–42 ng/mL and 30–235 ng/mL, respectively. The antitumor activity was tested in vivo in SCID mice bearing human OHS-M1 cells; TP-3(dsFv)_2_-PE38 showed a twofold increased effect compared to monovalent IT [95].

CD133 is a transmembrane glycoprotein, also known as AC133 or prominin-1, which is used as a cellular marker of cancer stem cells (CSCs) in many different malignancies, including sarcomas [96]. CSCs are usually a small subpopulation of cancer cells that are responsible for chemoresistance, relapsed disease and metastasis [97]. Unfortunately, normal stem cells, including hematopoietic, endothelial, and neuronal stem cells are CD133^+^ too. For this reason, new biotechnological strategies are fundamental to selectively kill CSCs, rescuing other CD133^+^ cells. Photochemical internalization is a site-specific and light-dependent drug delivery method that relies on the activation of a molecule, called photosensitizer, which co-localizes with the therapeutic agent of interest in endo-lysosomal compartments of the cells. The photosensitizer meso-tetraphenyl chlorin disulfonate (TPCS2a) was used to perform photochemical internalization of two anti-CD133 ITs. These ITs were obtained conjugating the biotinylated anti-CD133/1 (AC133) and anti-CD133/2 (293C) mAbs to streptavidin–saporin. The efficacy of this method was assessed on the undifferentiated human sarcoma cell line SW872, on the human FS cell line HT-1080 and on SW872-derived mouse xenografts cells, obtaining specific cytotoxic effects. Moreover, in vitro and in vivo experiments revealed a strong decrease in colony forming ability and a great tumor initiation inhibition of the surviving cells after photochemical internalization of the anti-CD133-saporin [98].

As discussed above, TEM1/endosialin/CD248 is a cell surface receptor highly expressed on human sarcomas that is considered a valid target for immunotherapeutic treatments. The human scFv-Fc fusion protein (78Fc) specifically bound TEM1^+^ sarcoma cell lines in vitro (SJSA-1, A673, MES-SA, and HOS) and sarcoma cells in xenografted nude mice. The 78Fc was chemically conjugated to the plant toxin saporin to augment its cytotoxicity. In vitro experiments revealed that 78Fc-Sap was able to specifically kill TEM^+^ sarcoma cells with a significantly higher effect in comparison with saporin alone. In vivo antitumor activity of 78Fc-Sap was assessed on SJSA-1 and A673 derived xenografts showing a high and specific tumor growth inhibition with no systemic toxicity even at the highest dose (0.2 mg/kg) [99].

As previously reported, the anti-endoglin mAb OMTX003 is a valid carrier to construct therapeutic ICs against ES. OMTX003 was also conjugated to nigrin b A chain. OMTX503 IT was highly stable, its cell surface binding ability was specific for endoglin^+^ cells and the cellular internalization was efficient. It showed an antiproliferative activity in vitro on three ES cell lines with different level of endoglin expression (RM82, TC71 and CADO) with IC_50_ values of 0.118 nM, 9.155 nM, and 17.38 nM, respectively. Thus, the cytotoxic effect was related to the endoglin expression level. OMTX503 was also tested in vivo on RM82-derived mouse xenografts at 0.5 mg/kg, obtaining good and significant results in terms of tumor growth inhibition and cell viability reduction [62].

ITs tested in preclinical studies are reported in Table 2.

### 3.3. Radioimmunoconjugates for Sarcoma

The first RIC approved in clinical practice was ibritumomab tiuxetan, in which the anti-CD20 mAb rituximab is (radio)labeled with Yttrium-90 for the treatment of non-Hodgkin’s lymphoma [100].

Despite most studies reported in literature describe the use of RICs for diagnostic purposes, some works depict the attempts to apply RIT to the treatment of sarcomas.

CD146 is a cancer associated cell adhesion molecule (CAM) overexpressed in several cancer types, including OS; it is associated with tumor progression, neoangiogenesis, and vascular development [101]. Anti-CD146 murine mAb OI-3 was (radio)labeled with Lutetium-177 or Iodine-125 and was tested in biodistribution/dosimetry experiments. Results showed promising data in terms of RIC tumor uptake in nude mice bearing OHS xenografts [102].

Insulin-like growth factor 2 receptor (IGF2R) is another valid target for OS treatment, because it is overexpressed in several cell lines and patient-derived OS cells [103]. A novel murine anti-IGF2R mAb, named 2G11, was (radio)labeled with Indium-111 to determine biodistribution and tumor uptake in OS tumor bearing SCID mice. Successively, 2G11 was (radio)labeled with both Lutetium-177 and Bismuth-213, obtaining good results in vivo in terms of slowing down tumor growth, without local or systemic toxicity referred [104].

Frizzled homologue 10 (FZD10) is the main target used for synovial sarcoma (SS) RIT. FZD10 is a transmembrane receptor of the Wnt signaling pathway whose gene is upregulated specifically in SS, but not expressed in any normal human tissue except for placenta [105]. The murine mAb 92–13 (radio)labeled with Yttrium-90 showed specific binding ability to FZD10 in vitro and in vivo, good internalization into FZD10^+^ cells and strong antitumor activity in SS mouse xenografts [106,107]. The humanized chimeric anti-FZD10 mAb OTSA101 was (radio)labeled with Indium-111 or Yttrium-90. In a phase 1 clinical trial, 20 patients with advanced/recurrent SS received an injection of the Indium-111-OTSA101 RIC to determine tumor uptake and biodistribution. Successively, only those patients (*n* = 10) that showed a significant tumor uptake, were treated with Yttrium-90-OTSA10. Unfortunately, no patients showed an objective tumor regression. In fact, best overall response was a stable disease in 3 patients [108]. Adsorbed dose simulations can explain tumor response on treated patients. The estimated biodistribution and dosimetry of (radio)labeled anti-FZD10, in normal tissue and tumor, was evaluated through Monte Carlo-based 3D simulations [109]. In a comparative preclinical study, OTSA101 was (radio)labeled with both Yttrium-90 and Astatine-211. Astatine-211-OTSA101, an α-emitting anti-FZD10 RIC, suppressed tumor growth of SS mouse xenografts more efficiently than the same dose of the Yttrium-90-OTSA101, a β-emitting anti FZD10 RIC, without remarkable toxic side effects [110]. This confirmed that α-RIT is superior to β-RIT in treating solid tumors because α-particles, with higher linear energy transfer, may have more advantages in terms of cytotoxicity compared to β-particles, with lower linear energy transfer [111].

The glycoprotein B7H3 is expressed on desmoplastic small round tumor cells (DSRCT), a rare sarcoma that affects adolescents and young adults involving the peritoneum. RIT treatment against DSRCT was tested in a phase 1 clinical trial (NCT01099644) on 52 patients. Murine anti-B7H3 mAb omburtamab (8H9) linked to Iodine-131 was administrated with an intraperitoneal injection. Related toxicity was mild and transient in almost all patients and adsorbed dose was low in normal tissues [112]. To date, Iodine-131-omburtamab is on a phase 2 clinical trial to improve patient survival (NCT04022213—recruiting status).

RICs tested in preclinical studies and clinical trials are reported in Table 3.

## 4. Conclusions

This review aims to provide a comprehensive overview of the latest advances in sarcoma immunotherapy and their impact on clinical oncology. The IC studies reported in this review show efficacy and clinical potential in sarcoma therapy. Although rare in adults, sarcomas are more frequent among pediatric tumors. Sarcomas are characterized by molecular and morphological complexity; the rarity and heterogeneity of sarcomas induce clinicians and researchers to seek and validate personalized therapeutic approaches. IC-based immunotherapy has been showing increasingly interesting results in terms of anti-tumor efficacy beside to a reduction of side effects. These positive results depend mainly on the possibility to select new engineered carrier moieties characterized by stability, binding specificity and reduced immunogenicity. The results obtained in preclinical studies with ICs in sarcoma models encouraged the translation from bench to bed.

The clinical studies over the last 20 years allowed nine ADCs to be approved by the FDA and many others are in phase 3 clinical trial [113] in different neoplastic diseases. Currently, seven ADCs and two RICs are under phase 1–2 clinical trials for sarcoma therapy and many other ICs have been evaluated in preclinical studies.

We believe that, in the near future, antibody-based therapeutic approaches could improve sarcoma patient outcome by overcoming some difficulties associated to standard therapy, such as the tumor resistance to the anticancer drugs, leading to patient relapse, and the onset of secondary malignancies [114,115].

## Figures and Tables

**Table 1:** Antibody–drug conjugates tested for sarcoma therapy.

ADC	Target	Antibody	Drug	Tumor	In Vitro	In Vivo	Clinical Trial	Ref.
anti-endosialin-MC-VC-PABC-MMAE	Endosialin	Fully human mAb	MMAE	ES, OS	√	√	-	[39]
ENDOS/ADC	Endosialin	hMP-E-8.3	Duocarmycin derivative	ES, OS	√	√	-	[40]
Glemtumumab vedotin	GPNMB	CR011	MMAE	OS	√	√	NCT02487979 Phase 2	[47]
ABBV-085	LRRC15	LRRC15 Ab1	MMAE	OS	√	√	NCT02565758 Phase 1	[49,50]
IMGN901	NCAM	N901	DM1	RMS, ES	√	√	NCT02452554 Phase 2	[57]
OMTX703	Endoglin	OMTX003	Cytolysin	ES	√	√	-	[62]
2h9-vc-MMAE	uPARAP	2h9	MMAE	FS, RMS	√	-	-	[67]
NBE-002	ROR1	humanized mAb	PNU-159682	S	-	√	NCT04441099 Phase 1/2	[69]
BA3021	ROR2	CAB	undisclosed	STS	√	√	NCT03504488 Phase 1/2	[72]
BA3011	AXL	CAB	undisclosed	S	√	√	NCT03425279 Phase 1/2	[76]
Enapotamab vedotin	AXL	human IgG1-κ	MMAE	S	√	√	NCT02988817 Phase 1/2	[77]
CD70-ADC	CD70	vorsetuzumab	MMAF	uLMS	√	√	-	[79]

The symbols √ and - mean tested and not tested, respectively, in vitro, in vivo or in a clinical trial. Abbreviations: ES, Ewing sarcoma; FS, fibrosarcoma; GPNMB, glycoprotein non-metastatic b; LRRC15, leucine-rich repeat containing 15; MMAE, monomethyl auristatin E; MMAF, monomethyl auristatin F; NCAM, neural cell adhesion molecule; OS, osteosarcoma; RMS, rhabdomyosarcoma; ROR, receptor tyrosine kinase-like orphan receptor; S, sarcoma (unspecified type); STS, soft tissue sarcoma; uLMS, uterine leiomyosarcoma; uPARAP, urokinase plasminogen activator receptor–associated protein.

**Table 2:** Immunotoxins tested for sarcoma therapy.

Target	Antibody	Toxin	Tumor	In Vitro	In Vivo	Clinical Trial	Ref.
CSPG4	αMCSP	ETA’	RMS	√	-	-	[82]
EGFR	425 (scFv)	ETA’	RMS	√	-	-	[86]
	murine mAb (clone 528)	Saporin	RMS	√	-	-	[87]
gp72	791T/36	RTA	OS	√	-	-	[89]
B7H3	8H9 (scFv)	PE38	OS	√	√	-	[92]
80 kDa sarcoma associated antigen	TP-3	PAP	OS	√	√	-	[94]
80 kDa sarcoma associated antigen	TP-3 (dsFv) TP-3 (dsFv)_2_	PE38	OS	√	√	-	[95]
CD133	AC133 293C	Saporin	S	√	√	-	[98]
TEM1	78Fc	Saporin	S	√	√	-	[99]
Endoglin	OMTX003	Nigrin-b A chain	ES	√	√	-	[62]

The symbols √ and - mean tested and not tested, respectively, in vitro, in vivo or in a clinical trial. Abbreviations: CSPG4, chondroitin sulfate proteoglycan 4; dsFv, disulfide-linked fragment variable; EGFR, epidermal growth factor receptor; ES, Ewing sarcoma; ETA’, truncated version of *Pseudomonas* exotoxin A; OS, osteosarcoma; PE, *Pseudomonas* exotoxin; RMS, rhabdomyosarcoma; RTA, ricin toxin A-chain; S, sarcoma (unspecified type); scFv, single-chain fragment variable.

**Table 3:** Radioimmunoconjugates tested for sarcoma therapy.

Target	Antibody	Radionuclide	Half-life	Emission	Tumor	In Vitro	In Vivo	Clinical Trial	Ref.
CD146	OI-3 CHOI-3.1 CHOI-3.3	^125^I ^177^Lu	59.5 days 6.7 days	Auger β^-^, Auger	OS	√	√	-	[102]
IGF2R	2G11	^111^In ^177^Lu ^213^Bi	67.4 h 6.7 days 46 min	γ β^-^, Auger α	OS	√	√	-	[104]
FZD10	92–13	^90^Y	64.1 h	β^-^	SS	√	√	-	[106,107]
FZD10	OTSA101	^111^In ^90^Y	67.4 h 64.1 h	γ β^-^	SS	-	√	Phase 1	[108,109]
FZD10	OTSA101	^90^Y ^211^At	64.1 h 7.2 h	β^-^ α, Auger	SS	-	√	-	[110]
B7H3	8H9	^131^I	8.0 days	β^-^	DSRCT	-	√	Phase 2	[112]

The symbols √ and - mean tested and not tested, respectively, in vitro, in vivo or in a clinical trial. Abbreviations: DSRCT, desmoplastic small round cell tumor; FZD10, frizzled homologue 10; IGF2R, insulin-like growth factor 2 receptor; OS, osteosarcoma; SS, synovial sarcoma.

## Data Availability

Not applicable.
